# Personal

**Published:** 1894-01

**Authors:** 


### Personal.

DR. ABRAHAM JACOBI, of New York, has been offered the Chair of Pediatrics in the University of Berlin, made vacant by the resignation of Professor Henoch. We are glad Dr. Jacobi has declined the honor, and will remain in New York to grace the Chair of Clinical Pediatrics in the College of Physicans and Surgeons, and to continue an ornament to the American medical profession. This is the first time such a distinction has been offered to an American physician. We take pleasure in printing the following editorial comment from the New York *Tribune*, of December 2, 1893:

It is a high and most unusual compliment which has been paid to Dr. Abraham Jacobi, of this city, in the invitation which lately came to him to assume a chair in the University of Berlin. While appreciating the compliment, New Yorkers cannot but rejoice that Dr. Jacobi promptly declined it. He has become so much attached to New York and to this country, that he feels his home to be here, and nothing apparently would induce him to make so radical a change as was proposed. He has lived here almost forty years, and has been an important factor in the community. We are glad that his eminent position in his profession has received this distinguished recognition, and glad that Dr. Jacobi is to remain one of our fellow-citizens ; and we are sure that this opinion will be shared by all who know him, either personally or by reputation.

---

Dr. Horace F. Clark, of Buffalo, at the meeting of the Section on Laryngology and Rhinology of the New York Academy of Medicine, December 27, 1893, reported a case of epithelioma of the larynx, and one of sarcoma (melanotic) of the nose.

---

Dr. J. W. Long, formerly of Randelman, N. C., has removed to Richmond, Va., and has been elected Professor of the Diseases of Women and Children in the Medical College of Virginia. Dr. Long will devote himself exclusively to the practice of his chosen specialty.

---

Dr. Seneca D. Powell, Professor of Surgery in the New York Post-Graduate Medical School and Hospital, was elected president of the Medical Society of the County of New York, at the annual meeting, held October 23, 1893.

---

Dr. Ernest Wende, Health Commissioner of Buffalo, has been elected a Fellow of the Royal Microscopical Society, London. This honor has been most appropriately conferred, in this instance, on a distinguished American student of microscopy.

---

Dr. Roswell Park, of Buffalo, whose severe illness from diphtheria we chronicled in November, has recovered, and is again at his post of duty. Dr. Park is to be congratulated upon his prompt restoration to comparatively good health after so serious a struggle with such a dangerous and even treacherous malady.

